# Complete chloroplast genome structural characterization of two *Phalaenopsis* (Orchidaceae) species and comparative analysis with their alliance

**DOI:** 10.1186/s12864-023-09448-5

**Published:** 2023-06-27

**Authors:** Lei Tao, Hanning Duan, Kaifeng Tao, Yan Luo, Qingqing Li, Lu Li

**Affiliations:** 1 Department of Biological Conservation, Southwest Forestry University, Kunming, Yunnan 650224 China; 2 Department of Life Science, Southwest Forestry University, Kunming, Yunnan 650224 China; 3 Department of Horticulture and Gardening, Xishuangbanna Tropical Botanical Garden, Chinese Academy of Sciences, Mengla, Yunnan 666303 China; 4 Kunming Xianghao Technology Co. Ltd., Kunming, Yunnan 650204 China

**Keywords:** *Phalaenopsis storbartiana*, *Phalaenopsis wilsonii*, Chloroplast genome, Orchidaceae, Structural characterization

## Abstract

**Background:**

The taxonomy and infrageneric delimitation of *Phalaenopsis* Blume has been significantly disputed due to some overlapping morphological features between species related, which needed further evidence for clarification. The structural characterization of complete chloroplast genomes of *P. storbatiana* and *P. wilsonii* were analyzed and compared with those of related taxa to provide a better understanding of their genomic information on taxonomy and phylogeny.

**Results:**

It was shown that chloroplast genomes of *Phalaenopsis storbatiana* and *P. wilsonii* had a typical quadripartite structure with conserved genome arrangements and moderate divergence. The chloroplast genomes of *P. storbatiana* and *P. wilsonii* were 145,885 bp and 145,445 bp in length, respectively, and shared a similar GC content of 36.8%. Gene annotations of two species revealed 109 single-copy genes consistently. In addition, 20 genes duplicated in the inverted regions, 16 genes each possessed one or more introns, and five *ndh* (NA (D)H dehydrogenase) genes were observed in both. Comparative analysis of the total cp genomes of *P. storbatiana* and *P. wilsonii* with those of other six related *Phalaenopsis* species confirmed the stable sequence identity for coding and non-coding regions and higher sequence variation in SC regions than IR regions. Most of their protein-coding genes had a high degree of codon preference. Moreover, 45 genes were discovered with significantly positive selection. However, different amplifications in IR regions were observed in these eight species. Phylogenetic analysis based on CDS from 60 species representing main clades in Orchidaceae indicated that *Phalaenopsis* species including *P. stobartiana* and *P. wilsonii* formed a monophyletic clade with high bootstrap nested in tribe Vandeae of Epidendroideae, which was consistent with those from previous studies.

**Conclusions:**

The results could provide insight into understanding the plastome evolution and phylogenetic relationships of *Phalaenopsis*.

**Supplementary Information:**

The online version contains supplementary material available at 10.1186/s12864-023-09448-5.

## Background

The broader genus *Phalaenopsis* Blume (Aeridinae, Vandeae, Epidendroideae, Orchidaceae) is consisted of about 40–45 species, which are collectively distributed from India to China, Korea, Japan, Thailand, Indochina, Malaysia, and Indonesia to the Philippines, Australia, and New Guinea [1]. There are 22 species recorded in China, including five endemic ones, which occurred in Southern China [2]. *Phalaenopsis wilsonii* Rolfe is endemic to China, while *P. stobartiana* Rchb.f. is distributed in South China and Burma [3]. Most *Phalaenopsis* species possess highly ornamental values and are used for breeding systems. However, the wild populations of *Phalaenopsis* have been decreasing due to their habitat fragmentation and over-exploration [1]. And then more attention should be paid to their biological conservation [2].

The phylogeny and infrageneric delimitation of *Phalaenopsis* has been disputed based on the morphological and molecular data available [4, 5]. *Phalaenopsis* was ever classified into two categories based on the presence of lip appendages [6] but into four groups according to the size of sepals and petals and the structure of the column and lip tip [7]. And then, it was divided into eight sections [8] or five subgenera with eight sections [9]. *Phalaenopsis wilsonii* and *P. stobartiana* were distinguished from other related species by flowers un-spurred [3]. Moreover, the intergeneric relationships were significantly confused with the alliance in the molecular phylogeny of Vandaeae investigated [10–13]. It was supported that a broad definition of *Phalaenopsis* was preferable, while a new infrageneric taxonomy encompassing four subgenera was proposed: *Parishianae* (H.R.Sweet) Christenson, *Phalaenopsis* (i.e., *Doritis* Lindl., *Kingidium* P.F.Hunt, and *Nothodoritis* Z.H.Tsi), *Hygrochilus* Pfitzer and *Ornithochilus* (Lindl.) Wall. ex Benth. [1, 14–16]. However, the phylogeny and taxonomy of *Phalaenopsis* have not been clear up to now. For example, it was suggested that the broad definition of *Phalaenopsis* should not include *Sedirea* Garay & H.R.Sweet [17, 18].

The chloroplast (cp) genome has been crucial in plant phylogenetics [19–21]. Compared with nuclear and mitochondrial gene sequences used in origin and phylogenetic relationships, the cp genomes are smaller, less prone to recombination, and have low rates of nucleotide substitutions [5, 22]. It has been frequently used in Orchidaceae for phylogenetics, which strongly supported the view that this family was comprised of five subfamilies [20, 22, 23]. It was implied that *Phalaenopsis* was related to *Neofinetia* Hu, *Pelatantheria* Ridl., and *Gastrochilus* D.Don and placed in Vandeae based on 79 CDS and four nrDNA from the cp genomic data [24]. Meanwhile, it seemed that some infrageneric relationships of *Phalaenopsis* had been stable. Subgenus *Aphyllae* (H.R.Sweet) Christenson consistently consisted of *P. wilsonii*, *P. stobartiana*, *P. honghenensis* F.Y.Liu, *P. minus* (Seidenf.), E.A. Christ., and *P. deliciosa* Rchb.f. based on a combined plastid sequence [9, 11, 12, 25]. However, *P. stobartiana* and *P. wilsonii* were also placed into subgenus *Parishianae* based on the broad definition of *Phalaenopsis* [2, 26].

Characterization of complete chloroplast genomes of *P. wilsonii* [27, 28] and *P. stobartiana* [25] were reported, but no additional genomic information was available. In this study, the structural and genomic information in detail were analyzed and compared with those of the related *Phalaenopsis* species downloaded from Genbank. The objectives of this study were: (1) to characterize and compare two complete chloroplast genome structures of *P. wilsonii* and *P. stobartiana* in detail, and (2) to provide further genomic information for a better understanding of phylogeny in *Phalaenopsis*.

## Results

### General data on the chloroplast genome

**Fig. 1:**
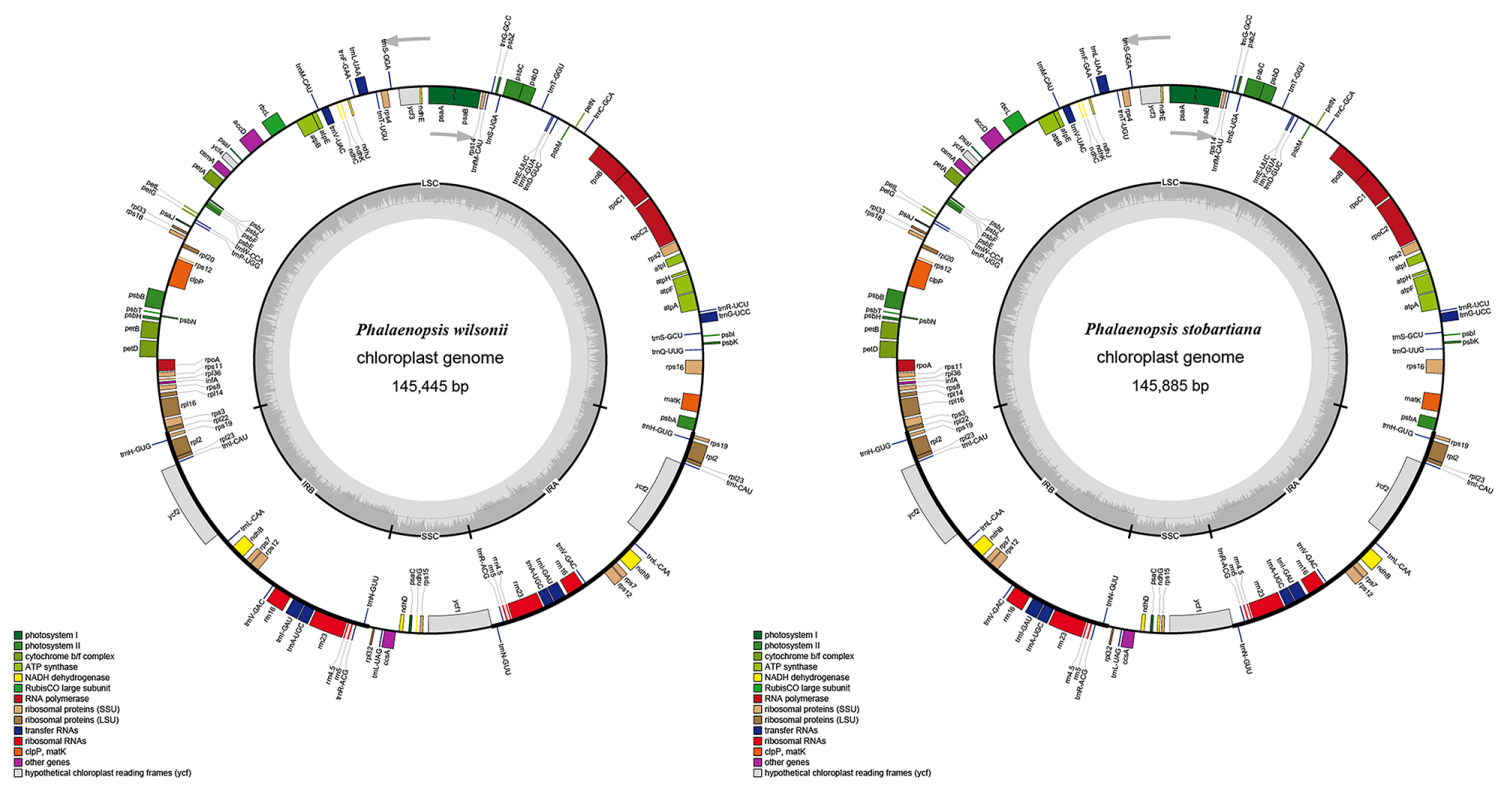
The chloroplast genome maps of *Phalaenopsis stobartiana* and *P. wilsonii*. Internal genes were clockwise transcribed, while external genes were counterclockwise transcribed. The inside circle bright and dark gray coloring indicated the genome guanine-cytosine (GC) content

The structures of chloroplast genomes of two *Phalaenopsis* species were highly similar. The total sizes of two cp genomes were 145,885 bp (*P. stobartiana*) and 145,445 bp (*P. wilsonii*) (Fig. 1; Table 1). Same as those of most angiosperms, their chloroplast genome structures displayed a typical quadripartite structure with a large single-copy (LSC) region (85,349 bp, 85,076 bp), a small single-copy (SSC) region (10,596 bp, 10,473 bp), and two inverted repeats (IR) regions (24,970 bp, 24,948 bp). In both cp genomes, the amounts of GC contents in LSC, SSC, and IR regions were 36.8%, 28.2%, and 43.3%, respectively. Comparative analysis of both cp genomes consistently showed that the GC content in IR regions was higher than in LSC and SSC regions. The GC content of the three positions of the two cp genomes was very similar. However, the third letter GC (29.72%) content was lower than the first (45.47%, 45.40%) and second (37.85%, 37.86%) letter GC content (Table 2). Both cp genomes contained 127 genes, including 82 CDS, eight rRNAs, and 37 tRNAs. However, the length of the LSC was different. It was longer in *Phalaenopsis stobartiana* than in *P. willsonii* (Table 1). Among these, there were 109 unique genes in each cp genome. The LSC region contained 63 CDS genes and 20 tRNA genes, whereas the SSC region comprised seven CDS genes and only one tRNA gene. Eight CDS (*ndhB*, *rpl2*, *rpl22*, *rpl23*, *rps7*, *rps12*, *rps19*, and *ycf2*), eight tRNA (*trnA-UGC*, *trnH-GUG*, *trnI-CAU*, *trnI-GAU*, *trnL-CAA*, *trnN-GUU*, *trnR-ACG*, and *trnV-GAC*), and four rRNA (*rrn4.5*, *rrn5*, *rrn16*, and *rrn23*) genes were repeated in the IR regions (Table S1). There were 16 genes with introns, 13 genes (*trnV-UAC*, *trnL-UAA*, *trnI-GAC*, *trnG-UCC*, *trnA-UGC*, *rps16*, *rpoC1*, *rpl2*, *rpl16, petD*, *petB*, *ndhB*, and *atpF*) of which had only one intron, while the others (*clpP*, *ycf3*, *rps12*) had two introns (Table S1). Four of the 16 intron-containing genes were in the IR regions, while 12 of the 16 genes spread across the LSC region. All the exons of tRNA genes in both segments were 20–50 bp in length. The *rpl16*, *petD*, and *petB* genes had one very short exon compared with other genes, while the *rpoC1* had one longer exon. In addition to the above, *rps12* was a unique trans-splicing gene in which the first exon dispersed in the LSC region, but the second and third exons were in IR regions. Five *ndh* (NA (D)H dehydrogenase) genes (*ndhB*/*C*/*D*/ *E*/*G*/*J*/*K*) were identified (Fig. 1, Table S2).

**Table 1:** The general genome characteristics of two *Phalaenopsis* species

Characteristics and Parameters	*P*.*stobartiana*	*P*.*wilsonii*
Total cp genome size (bp)	145,885	145,445
LSC length (bp)	85,349	85,076
SSC length (bp)	10,596	10,473
IR length (bp)	24,970	24,948
Total GC content (%)	36.8	36.8
GC content for LSC (%)	34.1	34.1
GC content for SSC (%)	28.2	28.2
GC content for IR (%)	43.3	43.3
Total number of genes	127	127
CDS genes	82	82
rRNAs genes	8	8
tRNAs genes	37	37

**Table 2:** The GC content of the three positions of two *Phalaenopsis* species

Species	1st letter GC	2nd letter GC	3rd letter GC
*P. stobartianna*	45.47%	37.85%	29.72%
*P. wilsonii*	45.50%	37.86%	29.72%

### Codon usage analysis

**Fig. 2:**
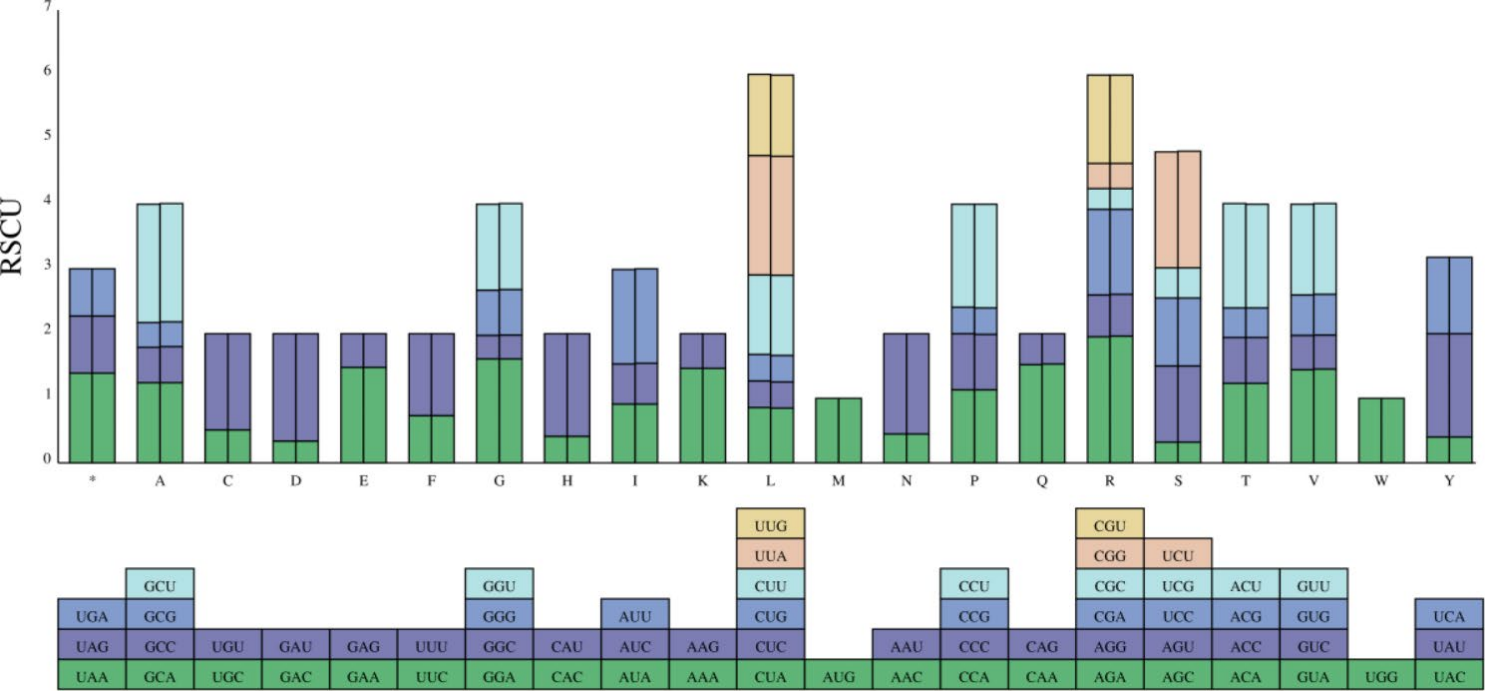
Codon content of 20 amino acids and stop codons in all protein-coding genes of the cp genomes of two *Phalaenopsis* species. The histogram above each amino acid shows codon usage within *Phalaenopsis*. Colors in the column graph reflected codons in the same colors shown below the figure. RSCU: relative synonymous codon usage; A: alanine; R: arginine; N: asparagine; D: asparagine; C: cysteine; Q: glutamine; E: glutamic; G: glycine; H: histidine; L: leucine; I: isoleucine; K: lysine; M: methionine; F: phenylalanine; P: proline; Ser: serine; T: threonine; W: tryptophan; Y: tyrosine; V: valine. Left: *Phalaenopsis wilsonii*; Right: *Phalaenopsis stobartiana*

Based on 82 coding sequences (CDS), codon usage frequency and relative synonymous codon usage (RSCU) were computed in both cp genomes. These CDS were composed of 23,281 (*Phalaenopsis wilsonii*) and 23,324 codons (*P*. *stobartiana*), respectively, and encoded 20 amino acids in the chloroplast genomes in them (Fig. 2, Table S3). The RSCU value of two chloroplast genomes was similar, with six codons for arginine (Arg) and leucine (Leu) and only one codon for methionine (Met) and tryptophan (Trp). Among them, leucine (Leu: 10.11%, 10.13%) was the amino acid that was utilized the most frequently, whereas cysteine (Cys: 1.19%, 1.19%) was the least ubiquitous amino acid in the two cp genomes. Except for methionine (Met) and tryptophan (Trp), practically all amino acids were encoded by 2–6 synonymous codons, according to the RSCU analysis. Relative synonymous codon usage was 1 for methionine (Met) and tryptophan (Trp). Thirty codons had RSCU > 1, and 31 had RSCU < 1. Almost CDS in *Phalaenopsis* species had the standard ATG start codon, but *rpl2* started with ATA/TAT. Among three stop codons, the TAA was the most common.

### Repeat sequences analysis

**Fig. 3:**
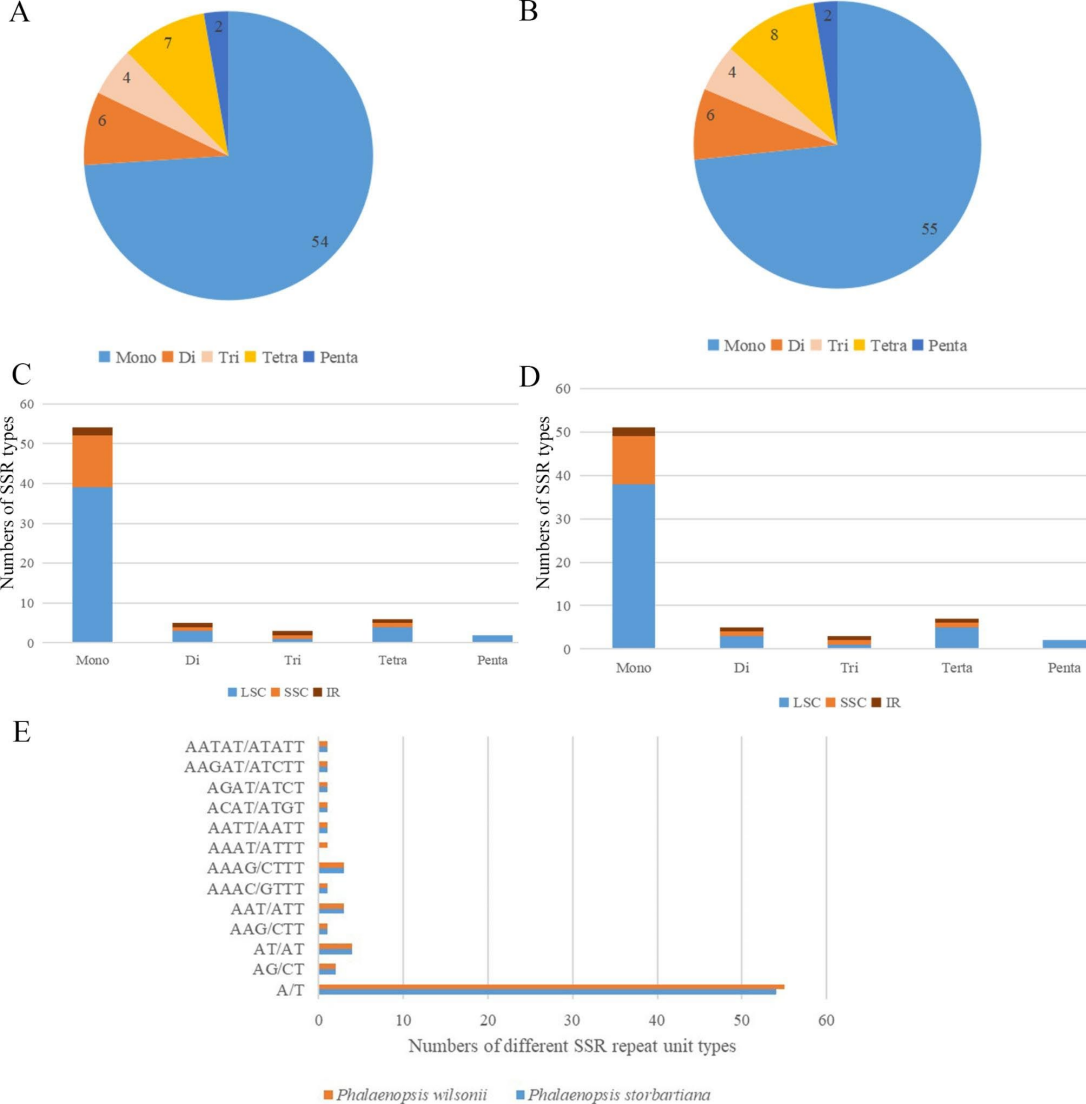
SSRs analysis of two chloroplast gnomes of *Phalaenopsis*. **A**: The number of SSRs distributed in different copy regions of *P. wilsonii*; **B**: The number of SSRs distributed in different copy regions of *P. stobartiana*; **C**: The number of SSR types of *P. wilsonii*; **D**: The number of SSR types of *P. stobartiana*; **E**: The number of different SSR repeat unit types

In this study, 75 (*Phalaenopsis stobartiana*) and 73 (*P. wilsonii*) SSRs were identified in two cp genomes, with 54–55 mononucleotides (mono-), six dinucleotides (di-), four trinucleotides (tri-), seven tetranucleotides (tetra-), and two pentanucleotides (penta-) (Fig. 3: A, B). Only the pentanucleotide was present in the IR regions, with most SSRs dispersed in the LSC, SSC, and IR regions. According to statistical analysis, most SSRs were in the LSC (51–54) region, while just 2 SSRs were dispersed in the IR regions (Fig. 3: C, D). Repeat units were composed mainly of A or T; besides, the mononucleotides of the two cp genomes were A/T type rather than G/C type. Furthermore, the AAAT/ATTT type tetranucleotide was only found in *P. willsoni*.

**Fig. 4:**
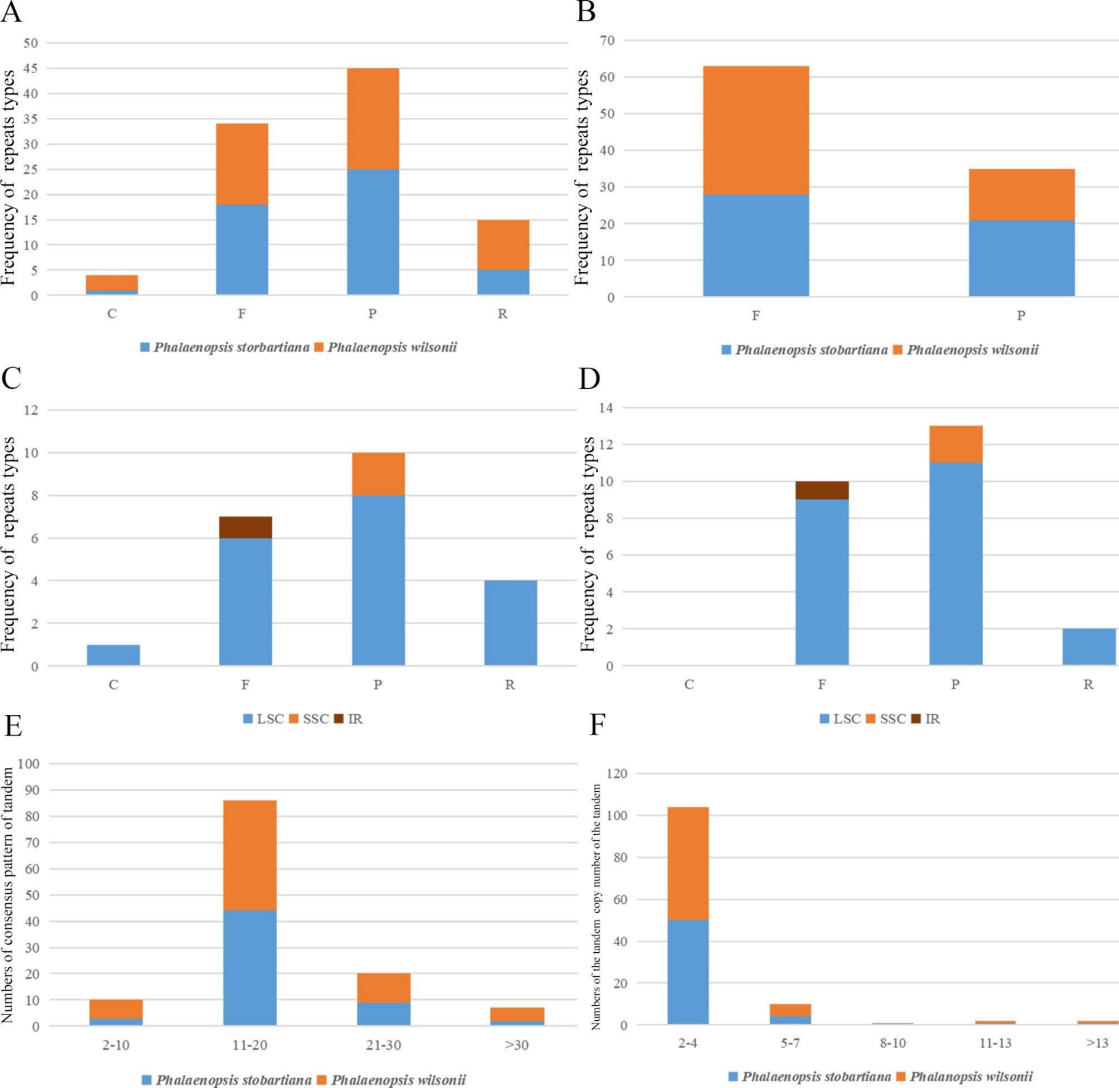
Analysis of repeat sequences of two chloroplast genomes of *Phalaenopsis*. **A**: The frequency of four repeats types of complete genome sequences; **B**: The frequency of repeats types of tandem sequences; **C**: The frequency of repeats types of *P. wilsonii*; **D**: The frequency of repeats types of *P. stobartiana*; **E**: The consensus pattern of tandem; **F**: The copy number of the tandem. Abbreviations: C, complement; F, forward; P, palindromic; R, reverse

Four different types of tandems were identified based on the complete genome sequence: complement (C), forward (F), palindromic (P), and reverse (R). Complete tandem content was the lowest, while the palindromic tandem content was the highest in the two cp genomes. However, the tandem sequence of the two cp genomes contained two different tandem forms (F and R) (Fig. 4: A, B). Except for *Phalaenopsis stobartiana*, there were almost tandem types in LSC region when comparing the two cp genomes. The F-type tandem was present in the LSC and IR regions; nevertheless, the P-type tandem also dispersed in the SSC region (Fig. 4: C, D). The consensus patterns in each of the two cp genomes ranged from two bp to more than 30 bp. Most of them were between 11 and 20 bp, according to the consensus pattern analysis (Fig. 4: E). The most tandem copy numbers of two cp genomes were between 2 and 4. However, there were no 8–10 copy numbers in the cp genome of *P. wilsonii* (Fig. 4: F). The tandem repeat sequence exhibited an enrichment of A/T nucleotides.

### IR expansion and contraction

**Fig. 5:**
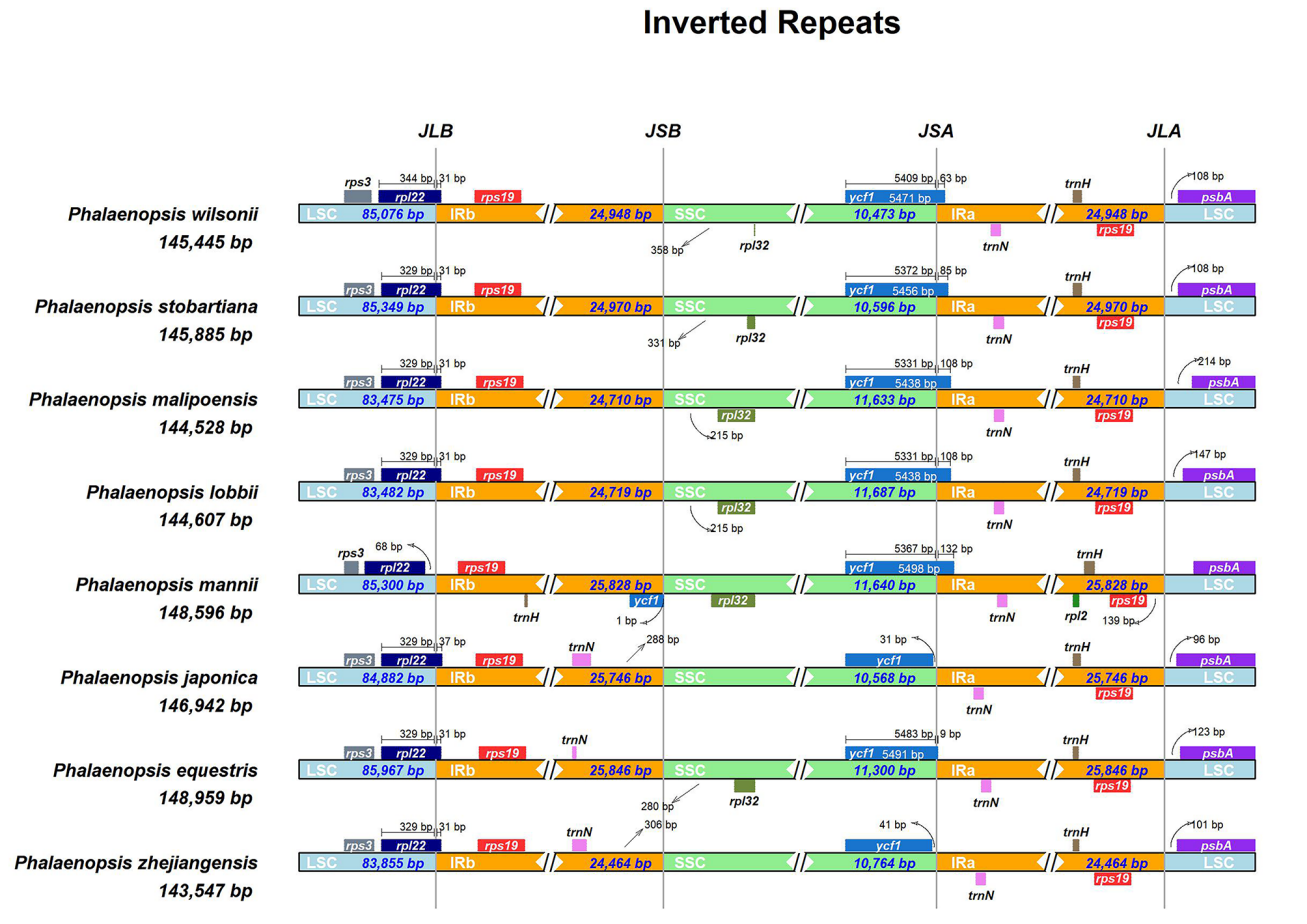
Comparison of the borders of LSC, SSC, and IR regions among eight sequenced Orchidaceae chloroplast genomes. Genes transcribed forward were shown above the lines, whereas genes transcribed reversely were shown below the lines. Gene lengths in the corresponding regions were displayed above the boxes of gene names. The number of bp represented by the arrow showed genes away from a specific region of the chloroplast genome. JLB (LSC/IRb), JSB (IRb/SSC), JSA (SSC/IRa), and JLA (IRa/LSC) denoted the junction sites between each corresponding two regions on the chloroplast genome

Comparative analysis between the two species investigated and six sibling ones, cp genomes of *Phalaenopsis* were highly conserved structurally. Nevertheless, some structural variations were observed on these boundaries (LSC/IRb, IRb/SSC, SSC/IRa, IRa/LSC) (Fig. 5). The *rps19-trnN-trnH* was in the junction of IRa/LSC region, while *rps19-trnN* was in the junction of IRb/SSC region in *P. japonica* (Rchb.f.) Kocyan & Schuit., *P. equestris* (Schauer) Rchb.f., and *P. zhejiangensis* (Z.H.Tsi) Schuit. Moreover, the *rps19-trnH* was only in the junction of IRb/LSC in *P. mannii* Rchb.f. The *rps3-rpl22* was in the junction of IRb/LSC. The *rpl22* was expanded from LSC to IRb region in eight species with 31 and 37 bp but distanced to IRb region with 68 bp in *P. mannii*. The *rpl32* was in the SSC region in six species, except *P. equestris* and *P. zhejiangensis*. Besides, the *rpl32* was the lowest in *P. wilsonii.* The *psbA* was in the LSC region. The *ycf1* was expanded from SSC to the IRa region in five species ranging from 9 to 132 bp, while it distanced to IRa region with 31 and 41 bp in *P. japonica* and *P. zhejiangensis*. In addition, the *ycf1* was also in the IRb region, and *rpl2* was in the IRa region in *P. manni*.

### Structural comparison and divergence hotspot identification analysis

**Fig. 6:**
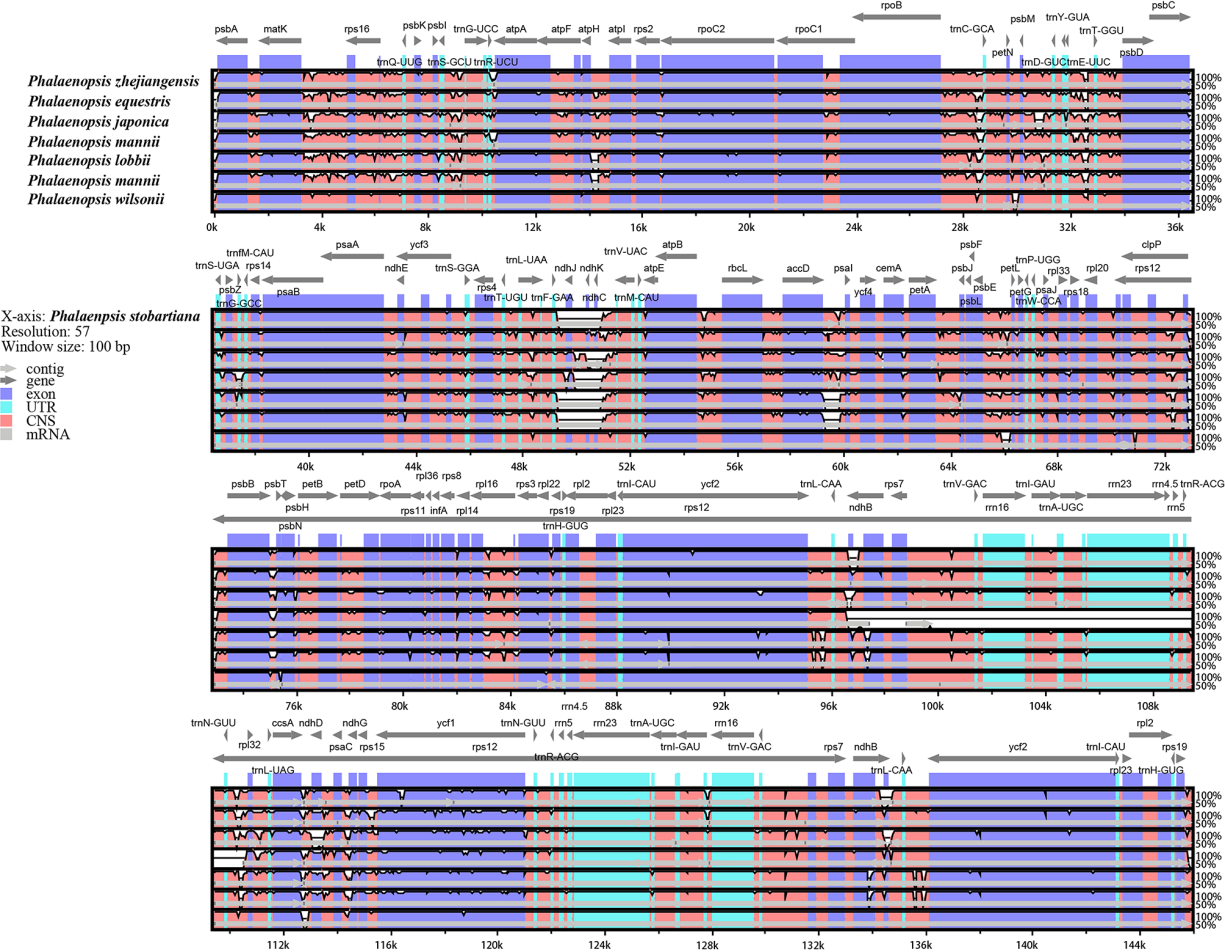
Sequence alignment of eight *Phalaenopsis* chloroplast genomes using mVISTA. The vertical scale indicates the percentage of identity, ranging from 50 to 100%. The horizontal axis indicated the coordinates within the chloroplast genome. Genome regions were color coded as exon, intron, and conserved non-coding sequences (CNS) and mRNA.

Based on the annotation of *Phalaenopsis stobartiana* as the reference, the chloroplast genome sequences of eight *Phalaenopsis* species were compared by mVISTA (Fig. 6). In comparison to LSC and SSC regions, the IR regions were more conversant. In contrast, the non-coding regions (CNS) were more diverse than the coding regions. The exons of the *ycf1* gene were the highest polymorphism. Moreover, the rRNA genes were highly conserved compared with other genes.

**Fig. 7:**
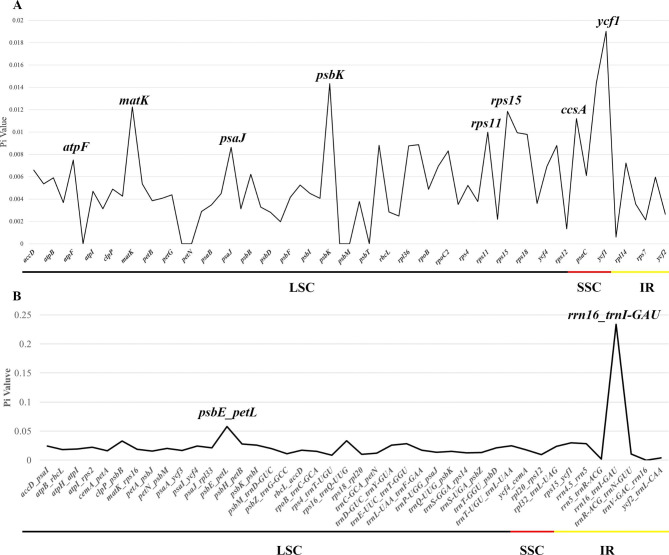
Sliding window analysis of eight cp genomes. (**A**) Comparison of the nucleotide variability (PI) among CDS regions; (**B**) Comparison of the nucleotide variability among IGS regions. X-axis: position of the midpoint of a window; Y-axis: nucleotide diversity of each window. The colored lines at the bottom delineate these gene locations in different regions

Examining CDS DNA polymorphism (Pi) revealed that the Pi value of LSC and SSC regions was greater than that of the IR regions, demonstrating that the latter were more varied. Three CDS stood out from the rest in terms of their higher Pi values: *matK* (0.01225), *psbK* (0.01434), and *ycf1* (0.01901) (Fig. 7:  A Table S5). There were two locations with high Pi values (> 0.05) for the IGS, including *psbE_petL* (0.05805) and *rrn16_trnI-GAU* (0.23387). The Pi value in IGS ranged from 0.00 to 0.23 with an average of 0.024 and from 0.00 to 0.019 with an average of 0.005938 in CDS (Fig. 7: B, Table S4).

### Positive selection analysis

The Bayes Empirical Bayes (BEB) method identified 45 significantly under positive selection genes, with the *psaJ*, *rps3*, *rps18*, and *ycf2* genes having two significant positive selection sites. Other genes had just one substantial positive selection site aside. The number of positive selections of genes in LSC and SSC regions was higher than in IR (Table 3, Table S6).

**Table 3:** The positive selection analysis of two *Phalaenopsis* species

M8							
Region	Gene name	Positive sites	Pr(ω > 1)	Region	Gene name	Positive sites	Pr(ω > 1)
LSC	*atpE*	135	1.000**	LSC	*psbM*	35	1.000**
LSC	*atpH*	82	1.000**	LSC	*psbN*	44	1.000**
LSC	*atpI*	248	1.000**	LSC	*psbT*	36	1.000**
SSC	*ccsA*	322	1.000**	LSC	*rbcL*	488	0.999**
LSC	*cemA*	230	1.000**	LSC	*rpl14*	123	1.000**
LSC	*infA*	78	1.000**	LSC	*rpl16*	136	1.000**
LSC	*petA*	321	1.000**	LSC	*rpl20*	118	1.000**
LSC	*petB*	216	1.000**	LSC	*rpl33*	67	1.000**
LSC	*petD*	164	1.000**	LSC	*rpl36*	38	1.000**
LSC	*petG*	38	1.000**	LSC	*rps3*	58 E	0.950*
LSC	*petL*	32	1.000**			219	1.000**
LSC	*petN*	30	1.000**	LSC	*rps4*	202	1.000**
LSC	*psaI*	37	1.000**	LSC	*rps8*	132	1.000**
LSC	*psaJ*	21 S	0.973*	LSC	*rps12*	124	1.000**
		45	1.000**	LSC	*rps14*	101	1.000**
LSC	*psbA*	354	1.000**	LSC	*rps18*	27 Q	0.964*
LSC	*psbB*	509	0.992**			102	1.000**
LSC	*psbC*	474	1.000**	LSC	*ycf4*	185	1.000**
LSC	*psbD*	354	1.000**	SSC	*rpl32*	58	1.000**
LSC	*psbE*	84	1.000**	IR	*rpl23*	94	1.000**
LSC	*psbF*	40	1.000**	IR	*rps7*	156	1.000**
LSC	*psbH*	74	1.000**	IR	*rps19*	93	1.000**
LSC	*psbI*	37	1.000**	IR	*ycf2*	562 I	0.969*
LSC	*psbJ*	41	1.000**			563 P	0.978*
LSC	*psbL*	39	0.999**			687 S	0.950*

*p > 95%; ** p > 99%

### Phylogenetic analysis

**Fig. 8:**
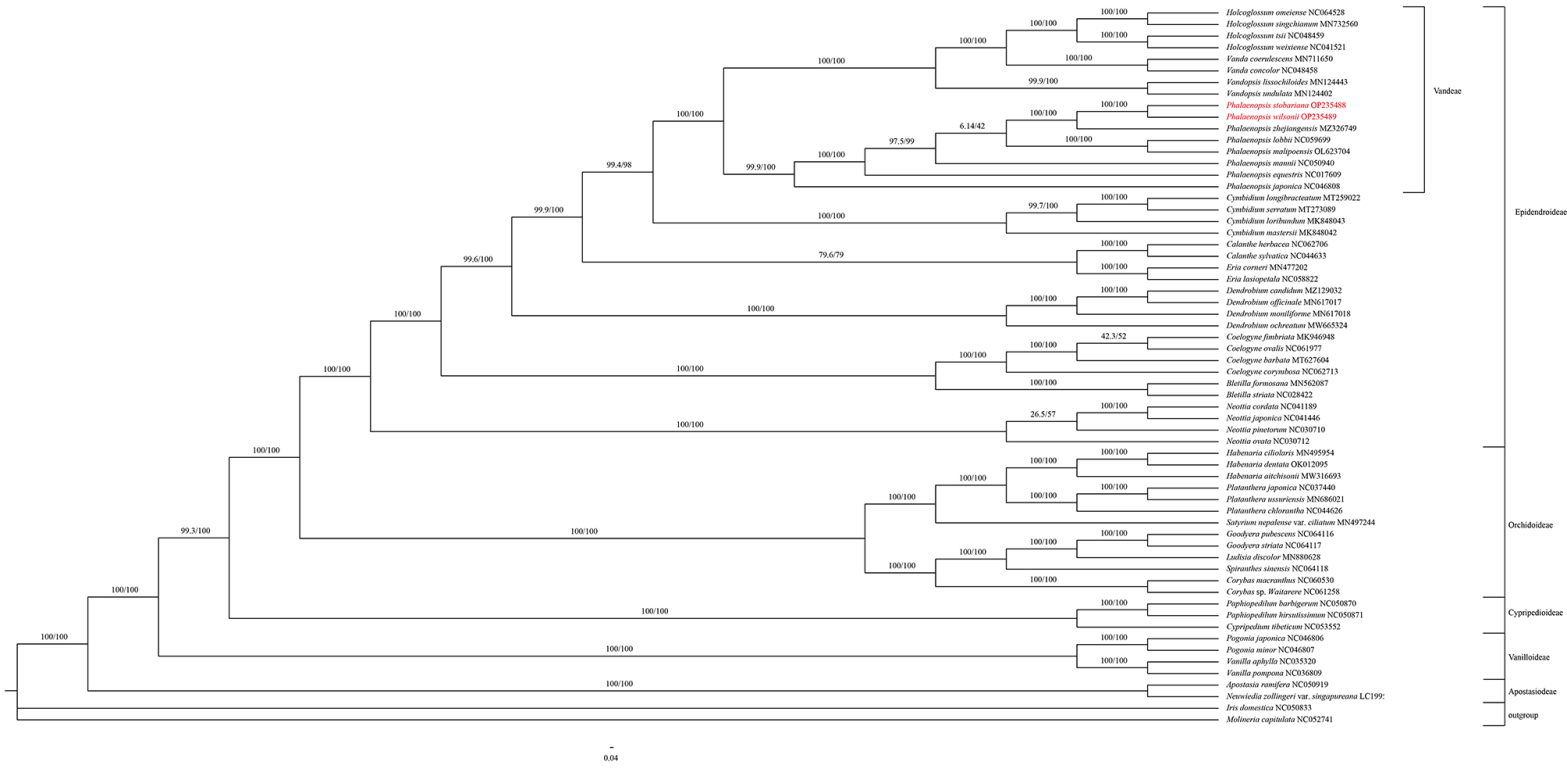
Phylogenetic tree reconstructed of Orchidaceae using Maximum-likelihood (ML) method based on 51 single-copy CDS sequences of 60 orchid species, with *Iris domestica* and *Molineria capitulata*as as outgroup

A Maximum-likelihood (ML) phylogenetic tree was constructed based on 51 single-copy CDS sequences of 60 species representing main clades in Orchidaceae, with *Iris domestica* (L.) Goldblatt & Mabb. and *Molineria capitulata* (Lour.) Herb. as outgroups, to shed fresh light on the phylogenetic position of *Phalaenopsis*. The ML tree (Fig. 8) showed that all taxon sampled formed five significant main clades corresponding to five subfamilies in Orchidaceae. Furthermore, two species investigated and six other taxa from *Phalaenopsis* were formed as a clade with strong support (with SH-aLRT: 99.9%, and UFBoot: 100%), which was related to *Vandopsis* Pfitzer, *Vanda* R.Br., and *Holcoglossum* Schltr. in the tribe Vandeae of subfamily Epidendroideae. It was indicated that *P. stobartiana* and *P. wilsonii* were grouped into a clade with strong support (with SH-aLRT: 100% and UFBoot: 100%).

**Fig. 9:**
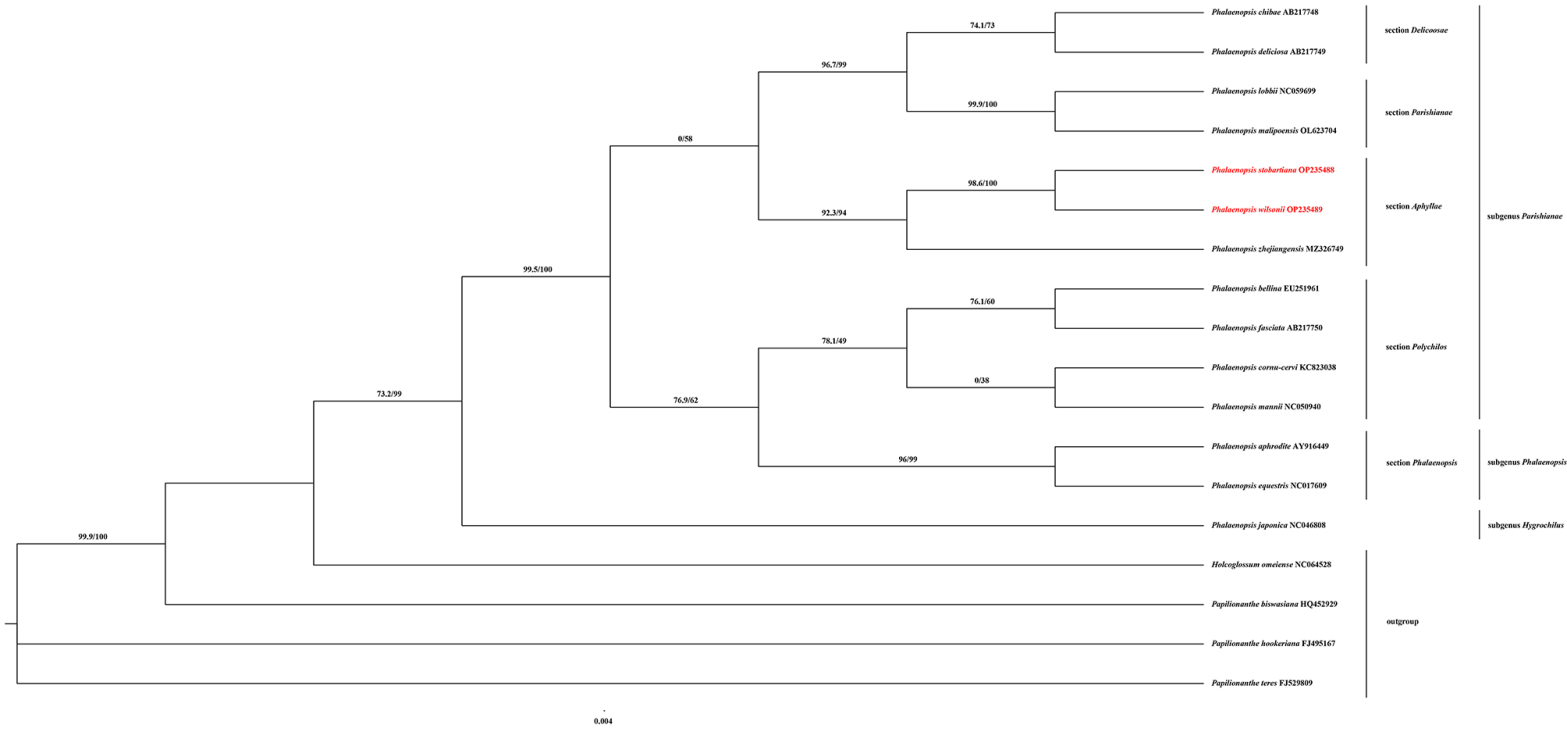
Maximum-likelihood phylogenetic tree of *Phalaenopsis* based on *matK* gene of 14 *Phalaenopsis* species, with four *Papilionanthe* species and one *Holcoglossum* species as outgroup

Considering the problematic taxonomy of *Phalaenopsis*, a phylogenetic tree was created using the Maximum-likelihood (ML) method based on the *matK* sequence of 14 *Phalaenopsis* species, with four *Papilionanthe* Schltr. species and a *Holcoglossum* species as the outgroups (Fig. 9). It was shown that *Phalaenopsis stobartiana* and *P. wilsonii*, which were related to *P. zhejiangensi*s, were assigned to the section *Aphyllae* subgenus *Parishianae* with strong support (SH-aLRT: 98.6% and UFBoot: 100%).

## Discussion

In this study, the complete chloroplast genomes of *Phalaenopsis stobartianna* and *P. wilsonii* were sequenced and compared with those of other related orchid species to learn more about the cp genomic information and the molecular phylogeny of *Phalaenopsis*.

The chloroplast genomes of *Phalaenopsis* were highly similar [27, 29–34], which was confirmed by new data presented here. The chloroplast genomes of *P. stobartiana* and *P. wilsonii* presented a typical quadripartite circular structure with the LSC and SSC regions divided by the IR regions, which were similar to the other orchids and most of the angiosperms with no significant differences [35, 36]. The genome size was different from the previous research, and 82 CDS were annotated in these two cp genomes, but 73, 74, and 76 CDS were annotated in previous research [25, 27, 28]. The annotation of the *ndh* CDS caused this difference. *P. wilsonii* and *P. stobartiana* contained *ndh B*, *C*, *D*, *E*, *G*, *J*, and *K*, while other *Phalaenopsis* species lacked *ndh* genes or *ndh* pseudogenes [24]. Eleven *ndh* genes in cp genomes encode the NAD(p)H dehydrogenase [37]. The Apostasioideae is *ndh*-complete, Vanilloideae is *ndh*-deleted, and Cypripedioideae, Orchidoideae, and Epidendroideae are both *ndh*-complete and *ndh*-deleted, indicating that a whole functioning set of the gene was present in the common ancestor of orchids, according to earlier research [38]. In some photoautotrophic plants, the *NDH* complex is unnecessary [38, 39].

In phylogenetic and relationship analysis, simple sequence repeats (SSRs), also known as microsatellites, are shorter tandem repeats made up of 1–6 bp repeat units widely dispersed across the chloroplast genome [40–43]. The most frequent SSRs in the chloroplast genomes of *Phalaenopsis storbartiana* and *P. wilsonii* were mononucleotide repeats. As well as other plants, the chloroplast SSRs are almost always composed of short poly-A or poly-T repeats, and the mononucleotide repeats are typically the most common forms [44–48]. Additionally, the GC content of IR regions was much higher than that of the LSC and SSC regions, and these characteristics were also revealed in other plant species [49, 50]. This phenomenon is caused by the presence of rRNA (*rrn4.5*, *rrn5*, *rrn16*, and *rrn23*) and tRNA genes in this region, which is the same as other Orchidaceae chloroplast genomes [40, 51–53].

Codon formation is necessary to convert genetic information from mRNA to protein [54], and codon bias, especially the third base usage pattern, is strongly connected [55]. It has been demonstrated that the GC composition influences the utilization of codons and amino acids and that the GC content of a third codon base (GC3) is thought to represent codon usage trends most closely [56]. Regarding *Phalaenopsis* species, the GC content was similar in this study but varies greatly among plant species [57]. The third-letter GC content of the two *Phalaenopsis* cp genomes was much lower than the first and second-letter GC content, and the findings were also reported in other plant species [58]. According to the RSCU analysis, six codons encoded leucine and arginine; however, only one codon encoded methionine and tryptophan, which was also reported in other Orichdaceae [40, 59].

The IR region of the chloroplast genome is thought to be the most conservative section. Nevertheless, its boundaries have frequently contracted and expanded related to the chloroplast genome evolution, which is the primary cause of the variation in chloroplast genome length [60–62]. In contrast to basal angiosperms and eudicots, most monocots have *trnH-rps19* clusters in each IR region [63]. However, in this study, the *trnH-rps19* clusters were only located in IRa region, which was consistent with *Platanthera ussuriensis* (Regel) Maxim. [64], and *Paphiopedilum henryanum* Braem [59]. The occurrence of the *trnH-rps19* gene cluster in the IR of most monocots has been claimed to be evidence of a duplication event that occurred before the divergence of monocot lineages, and fluxes in the IR borders have been suggested to implicate the taxonomic relationships among angiosperms [41, 63]. Furthermore, *Phalaenopsis zhejiangensis* and *P. japonica* were consistent with *P. aphrodite* Rchb.f. [34], of which the *ycf1* gene was only in the SSC region. In contrast, in other species, the *ycf1* gene spanned the SSC and IRa regions.

The divergent regions could offer valuable data for DNA barcoding and phylogenetic research, which are used as molecular markers in phylogenetic reconstruction studies [65, 66]. In this study, the nucleotide sequence of non-coding regions was more varied than the coding regions, which was generally consistent with other Orchidaceae chloroplast genomes [35, 40, 64]. Additionally, the CDS region analysis revealed that the genes *matK*, *psbK*, and *ycf1* had much higher Pi values. Of these, *matK* and *ycf1* have been employed as DNA markers for phylogenetic studies [66]. The *psbK* genes may be helpful for the phylogenetic analysis of chloroplast genomes in the NCBI database. In this research, *psbE_petL*, and *rrn16_trnI-GAU* also have the highest degree of variability, indicating a diversity of highly variable sequences in the chloroplast genome of orchids. However, *trnS_trnG, psaC_ndhE, clpP_psbB, rpl16* intron et al. were the highest degree of variability in *Phalaenopsis*, and *rpl32_trnL*, *trnE_trnT* et al. were the highest degree of variability in *Cymbidium* Sw. [67].

The ratio of substitution rates at synonymous and non-synonymous sites (dN/dS, ω) had been used to determine adaptive signals among species and infer the processes of evolution [68, 69]. Additionally, it could suggest that environmental factors impacted the evolution of chloroplast genomes, which was the primary cause of the divergence of many genes in cp chloroplast [70]. In this study, 45 genes were identified significantly under positive selection. Among them, *atpF*, *atpH*, *petL*, and *rps*4 genes were also found in other orchids [40, 71, 72]; the *atpE* and *petF* were also found in other plant species [73, 74]. Moreover, these genes could be used for identification and phylogenetic research for orchids.

The structural features of the chloroplast genome would aid in understanding plant phylogeny [30–32, 52, 53, 75]. Moreover, protein-coding regions and conserved sequences are informative for phylogeny and taxonomy [76]. It was confirmed that Orchidaceae was divided into five subfamilies, and *Phalaenopsis* species were grouped into a stable clade in Vandeae of Epidendroideae based on CDS presented here was consistent with previous data available [5]. In addition, the plastid *mat*K gene has been one of the most valuable single loci for plant phylogenetics at both shallow and deep stages of evolution [77–80].

*Phalaenopsis* was divided into five [9] or four subgenera in a broad definition of *Phalaenopsis* [1, 2, 15]. A ML phylogenetic tree based on *mat*K sequence from 14 *Phalaenopsis* species with related taxon in Vandeae as an outgroup presented here indicated *P. stobartiana* and *P. wilsonii* were grouped into a clade of section *Aphyllae*, subgenus *Parishianae*, together with a newly recorded species of *P. zhejiangensis*. It was congruent with the latest research on *Phalaenopsis* [2]. It seemed that *P. stobartiana* was more closely to *P. wilsonii* than *P. zhejiangensis*.

There were exhibited similar floral features in *Phalaenopsis stobartiana* and *P. wilsonii* by the presence of an inconspicuous spur and a nipple-shaped structure beneath the posterior callus [3]. However, they were distinguished by different mid-lobe of the labellum. The mid-lobe of the labellum was not obcordate without a terminal notch in *P. stobartiana*, but obcordate with an acentralapical fleshy knob in *P.wilsonii*[9].

The support of some internal nodes was low based on *matK* sequence (Fig. 9). Similarly, there were still some branches of the previous studies based on other plastid and/or nrDNA sequences that were also less supported [12, 26, 81]. The taxonomy and phylogeny of *Phalaenopsi*s remained unclear and needed to be clarified by more data [1, 2].

## Conclusion

Complete chloroplast genomes of *P. willsonii* and *P. stobartiana* were sequenced and analyzed, including the general genome structure, codon usage, repeat sequences, IR boundaries, DNA polymorphism, positive selection suites, and phylogenetic position. These cp genomic data were compared with those of the other six *Phalaenopsis* species available. It was confirmed that the cp genomic feature of *Phalaenopsis* was almost congruent and highly conserved, which could be used to understand the plastome evolution and evolutionary relationships of *Phalaenopsis*.

### Methods and materials

#### Ethical statement

No specific permits were required for the collection of specimens for this study. This research was carried out in compliance with the relevant laws of China.

#### Plant materials and chloroplast genome sequencing

Leaf samples of *P. stobartiana* (Cultivar No. 0020180019) and *P. wilosonii* (Cultivar No. 0020172683) were cultivated and obtained from the Xishuangbanna Tropical Botanical Garden, Chinese Academy of Sciences, Yunnan. The specimen was deposited in the Herbarium of Southwest Forestry University (HSFU, Lilu20180015, lilu@swfu.edu.cn). Total genomic DNA from fresh leaves was extracted by using the TiangenDNA kit (TIANGEN, China). An Illumina paired-end DNA library was constructed using the IlluminaTruSeq Library Preparation Kit (San Diego, CA, USA), following the manufacturer’s instructions. The library was sequenced by the Illumina Hiseq 2500 sequencing platform (Illumina, CA, USA) at Personal Biotechnology Co., Ltd (Shanghai, China).

#### Chloroplast genome assembly and annotation

The two complete chloroplast genome from the clean reads was assembled by the GetOrganelle version 1.7.7.0 [82] and annotated the new sequences using the Geneious Prime version 2020.0.4 [83]. The complete chloroplast genomes sequences of *P. stobartiana* and *P. wilsonii* were submitted to GenBank (Accession number: OP235488 and OP235489). The circular genome maps were drawn by the OGDRAW program (https://chlorobox.mpimp-golm.mpg.de/OGDraw) [69].

#### Sequence analysis and statistics

The repeat sequences were analyzed by REPuter (https://bibiserv.cebitec.uni-bielefeld.de/reputer/) [84], which included forward (F), reverse (R), complement (C) and palindromic (P) repeat with maximal repeat size set to 50 bp, minimal repeat size set to 30 bp, and hamming distance set to 8 [31]. In addition to the above, the tandem repeat sequences were detected by Tandem Repeats Finder with default parameters (http://tandem.bu.edu/trf/trf.html) [85]. By setting the minimum number of repeats to 10, 5, 4, 3, and 3 for mononucleotide (mono-), dinucleotide (din-), trinucleotide (tri-), tetranucleotide (tetra-), pentanucleotide (penta-), and hexanucleotide (hexan-), respectively, simple sequence repeats (SSR), a tract of repetitive DNA that typically ranges in length from 1 to 6 nucleotides, were detected by via MISA (https://webblast.ipk-gatersleben.de/misa/index.php?action=1) [86, 87]. Codon usage was analyzed by MEGA11 software [88], and the relative synonymous codon usage (RSCU) and amino acid frequencies were calculated with default settings [89]. The RSCU analysis was performed using JSHYCloud (http://cloud.genepioneer.com:9929). In addition, the GC content of the three positions was analyzed by CUSP on EMBOSS program (http://emboss.toulouse.inra.fr/cgi-bin/emboss/cusp) [90].

#### Sequence divergence and genome comparison

The pairwise alignments and sequence divergence of *Phalaenopsis wilsonii* and *P. stobartiana* with six other *Phalaenopsis* species were performed by the mVISTA with Shuffle-LAGAN mode (https://genome.lbl.gov/cgi-bin/VistaInput?num_seqs=2) [91]. Using the web tool IRSCOPE (https://irscope.shinyapps.io/irapp/), the contraction and extension of the IR borders between the four major areas (LSC/IRa/SSC/IRb) of the eight chloroplast genome sequences were performed [92].

#### Positive selection analysis

The CDS sequences were extracted by PhyloSuite version 1.2.2 [93], and the single-copy CDS sequences were aligned by MAFFT version 7 [94]. The phylogenetic tree based on CDS was platformed by MEGA 11 with Neighbor-Joining (NJ) methods [88]. The non-synonymous (dN) and synonymous (dS) substitution rates were calculated by the CodeML algorithm implemented in EasyCodeML [95] and selected the M8 mode for selection suites to detect the protein-coding genes under selection in the eight *Phalaenopsis* species.

#### Phylogenetic analysis

Sixty complete chloroplast genomes were chosen to build phylogenetic trees to determine the position of *P. wilsonii* and *P. stobartiana* within Orchidaceae. The complete chloroplast genome sequences of 58 orchid species were downloaded from the NCBI database, representing all five subfamilies of Orchidaceae (Orchidoideae, Epidendroideae, Cypripediordeae, Vanilloideae, and Apostasioideae). As Orchidaceae were sister to all other Asparagles [96, 97], two species from Iridaceae (*Iris domestica* (L.) Goldblatt & Mabb.) and Hypoxidaceae (*Molineria capitulata* (Lour.) Herb.) were selected as outgroups. These single-CDS sequences (Table S7) were extracted by PhyloSuite version 1.2.2 [93], aligned by MAFFT version 7 [94], trimmed by Gblocks [98], and concatenated by plugins in PhyloSuite version 1.2.2 [93]. The Maximum-Likelihood (ML) tree was performed in GTR + F + R3 mode based on CDS sequences by IQ-TREE 2 with 5000 ultrafast bootstrap (UFBoot) and 5000 SH-aLRT [99–101]. The 14 *matK* gene sequences, marked complete CDS, were downloaded from the NCBI database, extracted by PhyloSuite version 1.2.2 [93], and trimmed by Gblocks [98]. The phylogenetic tree based on *matK* gene sequence was constructed by IQ-TREE 2 in K3Pu + F + G4 mode, with 5000 ultrafast bootstrap (UFBoot) and 5000 SH-aLRT [99–101], with four *Papilionanthe* species and a *Holcoglossum* species as outgroups. The taxonomic system was adopted based on the broad definition of *Phalaenopsis*^2018^ [2].

## Electronic supplementary material

Below is the link to the electronic supplementary material.

Supplementary Material 1

Supplementary Material 2

Supplementary Material 3

Supplementary Material 4

Supplementary Material 5

Supplementary Material 6

## Data Availability

The datasets generated or analyzed during the current study are available in the NCBI BioProject (PRJNA861671, SRA SRR20710655, and SRA20710656).
