# Supplementary material for: Complete chloroplast genome structural characterization of two Phalaenopsis (Orchidaceae) species and comparative analysis with their alliance
Source: BMC Genomics. 2023 Jun 27;24:359. doi: 10.1186/s12864-023-09448-5 (PMC10294358; doi:10.1186/s12864-023-09448-5)
Supplement: Supplementary file 1 — Supplementary Material 1 [file 12864_2023_9448_MOESM1_ESM.doc]

| Table S1. Genes annotation for the chloroplast genome of the two Phalaenopsis species | | |
| --- | --- | --- |
| Categories of genes | Groups of genes | Name of genes |
| Genetic genes | Large subunit of ribosome (LSU) | *rpl2**(x2), rpl14, rpl16*, rpl20, rpl22, rpl23(x2), rpl32, rpl33, rpl36 |
| System | Small subunit of ribosome (SSU) | *rps2*,rps3,rps4,rps7(x2), rps8, rps11, rps12**(x2),rps14, rps15, rps16, rps18, rps19(x2) |
|  | RNA polymerase | *rpoA*, rpoB, rpoC1, rpoC2 |
|  | Ribosomal RNA (rRNA) | *rrn4.5*(x2), rrn5(x2), rrn16(x2), rrn23(x2) |
|  | Transfer RNA (tRNA) | *trnA-UGC**(x2), trnC-GCA, trnD-GUC, trnE-UUC,  trnF-GAA, trnfM-CAU, trnG-GCC*, trnG-UCC, trnH-GUG(x2), trnI-CAU(x2), trnI-GAU*(x2), trnL-CAA(x2), trnL-UAA*, trnL-UAG, trnM-CAU, trnN-GUU(x2), trnP-UGG, trnQ-UUG, trnR-ACG, trnR-UCU, trnS-GGA, trnS-UGA, trnT-GGU, trnT-UGU, trnV-GAC(x2), trnW-CCA, trnY-GUA |
|  | Translational initiation factor | *infA* |
| Photosynthetic genes | Photosystm I | *psaA*, psaB, psaC, psaI, psaJ |
|  | Photosystem II | *psbA*, psbB, psbC, psbD, psbE, psbF, psbH, psbI, psbJ, psbK, psbL, psbM, psbN, psbT, psbZ |
|  | NADH dehydrogenase | *ndhB*(x2), ndhC, ndhD, ndhE, ndhG, ndhJ, ndhK |
|  | Cytochrome b/f complex | *petA*, petB*, petD*, petG, petL, petN |
|  | ATP synthase | *atpA*, atpB, atpE, atpF*, atpH, atpI |
|  | Large subunit of Rubisco | *rbcL* |
| Biosynthesis genes | Maturase | *matK* |
|  | ATP-dependent protease proteolytic  subunit | *clpP*** |
|  | Cov envolpe membrane protein | *cemA* |
|  | Acetyl-CoA-carboxylase | *accD* |
|  | C-type cytchrome synthesis gene | *ccsA* |
|  | Hypothetic chloroplast reading frames | *ycf1*, ycf2(x2), ycf3**, ycf4 |
| *: contains one intron; **: contains two introns; (×2): genes located in IRs | | |

| Table S2. The genes with introns and the lengths of exons and intron | | | | | | |
| --- | --- | --- | --- | --- | --- | --- |
| Gene | Location | Exon Ⅰ(bp) | Intron Ⅰ (bp) | Exon Ⅱ (bp) | Intron Ⅱ (bp) | Exon Ⅲ (bp) |
| *clpP* | LSC | 252 | 684 | 292 | 1022 | 146 |
| *ycf3* | LSC | 153 | 739 | 228 | 721 | 126 |
| *rps12* | LSC | 114 | - | 26 | 548 | 232 |
| *trnV-UAC* | LSC | 35 | 582 | 39 |  |  |
| *trnL-UAA* | LSC | 35 | 732 | 50 |  |  |
| *trnI-GAC* | IR | 35 | 951 | 37 |  |  |
| *trnG-UCC* | LSC | 23 | 694 | 48 |  |  |
| *trnA-UGC* | IR | 35 | 802 | 38 |  |  |
| *rps16* | LSC | 40 | 944 | 245 |  |  |
| *rpoC1* | LSC | 453 | 2343 | 1608 |  |  |
| *rpl2* | IR | 431 | 664 | 385 |  |  |
| *rpl16* | LSC | 9 | 191 | 399 |  |  |
| *petD* | LSC | 8 | 840 | 484 |  |  |
| *petB* | LSC | 6 | 728 | 642 |  |  |
| *ndhB* | IR | 108 | 412 | 714 |  |  |
| *atpF* | LSC | 148 | 978 | 410 |  |  |
