# Supplementary material for: Complete chloroplast genome structural characterization of two Phalaenopsis (Orchidaceae) species and comparative analysis with their alliance
Source: BMC Genomics. 2023 Jun 27;24:359. doi: 10.1186/s12864-023-09448-5 (PMC10294358; doi:10.1186/s12864-023-09448-5)
Supplement: Supplementary file 2 — Supplementary Material 2 [file 12864_2023_9448_MOESM2_ESM.doc]

| Table S3 RSCU of two *Phalaenopsis* species | | | | | |
| --- | --- | --- | --- | --- | --- |
| **AA** | Codon | *Phalaenopsis wilsonii* | | *Phalaenopsis stobartiana* | |
|  |  | Count | RSCU | Count | RSCU |
| **Phe (F)** | UUU(F) | 833 | 1.27 | 839 | 1.27 |
|  | UUC(F) | 479 | 0.73 | 479 | 0.73 |
| **Leu (L)** | UUA(L) | 720 | 1.84 | 723 | 1.84 |
|  | UUG(L) | 494 | 1.26 | 495 | 1.26 |
|  | CUU(L) | 484 | 1.23 | 487 | 1.24 |
|  | CUC(L) | 159 | 0.41 | 159 | 0.4 |
|  | CUA(L) | 337 | 0.86 | 336 | 0.85 |
|  | CUG(L) | 160 | 0.41 | 162 | 0.41 |
| **Ile (I)** | AUU(I) | 947 | 1.46 | 947 | 1.46 |
|  | AUC(I) | 404 | 0.62 | 405 | 0.63 |
|  | AUA(I) | 590 | 0.91 | 592 | 0.91 |
| **Val (V)** | GUU(V) | 434 | 1.4 | 435 | 1.4 |
|  | GUC(V) | 164 | 0.53 | 164 | 0.53 |
|  | GUA(V) | 449 | 1.44 | 451 | 1.45 |
|  | GUG(V) | 197 | 0.63 | 197 | 0.63 |
| **Pro (P)** | CCU(P) | 374 | 1.59 | 376 | 1.6 |
|  | CCC(P) | 204 | 0.87 | 202 | 0.86 |
|  | CCA(P) | 265 | 1.13 | 265 | 1.13 |
|  | CCG(P) | 96 | 0.41 | 96 | 0.41 |
| **Thr (T)** | ACU(T) | 468 | 1.61 | 467 | 1.6 |
|  | ACC(T) | 206 | 0.71 | 207 | 0.71 |
|  | ACA(T) | 358 | 1.23 | 360 | 1.23 |
|  | ACG(T) | 134 | 0.46 | 134 | 0.46 |
| **Ala (A)** | GCU(A) | 546 | 1.83 | 545 | 1.83 |
|  | GCC(A) | 165 | 0.55 | 166 | 0.56 |
|  | GCA(A) | 368 | 1.24 | 368 | 1.24 |
|  | GCG(A) | 112 | 0.38 | 112 | 0.38 |
| **Try (Y)** | UAU(Y) | 647 | 1.6 | 647 | 1.6 |
|  | UAC(Y) | 162 | 0.4 | 164 | 0.4 |
| **His (H)** | CAU(H) | 469 | 1.59 | 471 | 1.59 |
|  | CAC(H) | 122 | 0.41 | 123 | 0.41 |
| **Gln (Q)** | CAA(Q) | 665 | 1.52 | 665 | 1.53 |
|  | CAG(Q) | 208 | 0.48 | 207 | 0.47 |
| **Asn (N)** | AAU(N) | 874 | 1.55 | 874 | 1.55 |
|  | AAC(N) | 254 | 0.45 | 255 | 0.45 |
| **Lys (K)** | AAA(K) | 989 | 1.46 | 991 | 1.46 |
|  | AAG(K) | 369 | 0.54 | 370 | 0.54 |
| **Asp (D)** | GAU(D) | 805 | 1.66 | 805 | 1.66 |
|  | GAC(D) | 162 | 0.34 | 162 | 0.34 |
| **Glu (E)** | GAA(E) | 959 | 1.48 | 959 | 1.48 |
|  | GAG(E) | 336 | 0.52 | 336 | 0.52 |
| **Cys (C)** | UGU(C) | 207 | 1.49 | 207 | 1.49 |
|  | UGC(C) | 70 | 0.51 | 70 | 0.51 |
| **Arg (R)** | CGU(R) | 335 | 1.37 | 335 | 1.37 |
|  | CGC(R) | 77 | 0.32 | 77 | 0.32 |
|  | CGA(R) | 321 | 1.32 | 320 | 1.31 |
|  | CGG(R) | 95 | 0.39 | 95 | 0.39 |
|  | AGA(R) | 477 | 1.95 | 479 | 1.96 |
|  | AGG(R) | 159 | 0.65 | 159 | 0.65 |
| **Gly (G)** | GGU(G) | 507 | 1.33 | 508 | 1.33 |
|  | GGC(G) | 139 | 0.36 | 140 | 0.37 |
|  | GGA(G) | 614 | 1.61 | 615 | 1.61 |
|  | GGG(G) | 266 | 0.7 | 266 | 0.7 |
| **Ser (S)** | UCU(S) | 545 | 1.79 | 547 | 1.8 |
|  | UCC(S) | 320 | 1.05 | 320 | 1.05 |
|  | UCA(S) | 359 | 1.18 | 361 | 1.18 |
|  | UCG(S) | 143 | 0.47 | 143 | 0.47 |
|  | AGU(S) | 359 | 1.18 | 359 | 1.18 |
|  | AGC(S) | 97 | 0.32 | 98 | 0.32 |
| **Trp (W)** | UGG(W) | 408 | 1 | 410 | 1 |
| **Met (M)** | AUG(M) | 533 | 1 | 535 | 1 |
| **Stop (*)** | UAA(*) | 38 | 1.39 | 38 | 1.39 |
|  | UAG(*) | 24 | 0.88 | 24 | 0.88 |
|  | UGA(*) | 20 | 0.73 | 20 | 0.73 |
